# Amantadine Reshapes Brain Oscillatory Dynamics in Pediatric Disorders of Consciousness

**DOI:** 10.1155/np/2959425

**Published:** 2026-07-13

**Authors:** Mengqing Luo, Rui Sun, Xuanhui Ren, Juan Cheng, Meng Zhang, Huaqian Wang, Ruihua Xu, Jun Tan, Jialei Ge, Xu Zhang, Yan Ma, Chong Lu

**Affiliations:** ^1^ Department of Rehabilitation Medicine, Traditional Chinese and Western Medicine Hospital of Wuhan, Tongji Medical College, Huazhong University of Science and Technology, Wuhan, China, hust.edu.cn; ^2^ Department of Pediatrics, Renmin Hospital of Wuhan University, Wuhan, China, rmhospital.com; ^3^ College of International Education, Minzu University of China, Beijing, China, muc.edu.cn; ^4^ Department of Rehabilitation Medicine, Tongji Hospital, Tongji Medical College, Huazhong University of Science and Technology, Wuhan, China, hust.edu.cn

**Keywords:** amantadine, cortical oscillations, covert consciousness, disorders of consciousness, EEG, pediatric brain injury

## Abstract

**Background:**

Disorders of consciousness (DoC) in children following severe brain injury remain a major clinical challenge, and pharmacological interventions with objective neurophysiological evidence are limited. Amantadine has shown potential benefits in adult DoC, but its neural effects in pediatric populations remain poorly understood. This study investigated whether amantadine treatment is associated with changes in resting‐state electroencephalography (EEG) oscillatory dynamics in children with DoC.

**Methods:**

Thirty participants were enrolled, including 15 children with DoC and 15 age‐ and sex‐matched typically developing (TD) controls. The DoC group received oral amantadine for 1 month at weight‐adjusted doses. Resting‐state EEG was recorded at baseline and after treatment. Relative EEG amplitude (RelAmp) was calculated for five frequency bands (delta, theta, alpha, beta, and gamma), and two spectral ratios—the alpha–theta ratio (ATR) and the slow–fast frequency ratio (SFR)—were derived to characterize the balance between slow and fast oscillatory activity. Behavioral responsiveness was assessed using the Coma Recovery Scale–Revised (CRS‐R).

**Results:**

At baseline, children with DoC showed significantly lower ATR, SFR, alpha amplitude, and gamma amplitude than TD controls, indicating a shift toward slower cortical oscillatory activity. ATR, SFR, and alpha amplitude were positively correlated with CRS‐R scores, suggesting that faster oscillatory dynamics were associated with better behavioral responsiveness. Following amantadine treatment, ATR, alpha amplitude, and gamma amplitude increased significantly within the DoC group, reflecting a shift toward faster cortical oscillatory activity. Notably, these EEG changes preceded overt behavioral improvement: CRS‐R scores did not change significantly immediately after treatment but improved significantly at the 3‐month follow‐up. This temporal dissociation suggests that neurophysiological recovery may emerge earlier than overt behavioral responsiveness and may be relevant to covert recovery processes in pediatric DoC.

**Conclusion:**

Amantadine treatment was associated with selective modulation of cortical oscillatory dynamics in children with DoC. EEG spectral measures may provide objective biomarkers for detecting early neural recovery and monitoring longitudinal brain changes in pediatric DoC.

## 1. Introduction

Children who survive acquired brain injury may remain in prolonged disorders of consciousness (DoC), yet pediatric DoC remains a markedly under‐studied and clinically consequential condition. Studying this population is critically important not only because affected children may live for many years with profound disability, but also because consciousness assessment and recovery in childhood unfold against a background of ongoing brain maturation. This developmental context creates a double challenge: on the one hand, heightened neuroplasticity may confer substantial potential for recovery; on the other hand, age‐dependent variability in motor, language, and social behaviors can obscure the detection of residual consciousness and complicate prognostic judgments. Behavioral tools developed for adults—such as the Coma Recovery Scale–Revised (CRS‐R)—therefore, have limited validity in young, nonverbal, or developmentally immature children. Slomine et al. [1] developed a pediatric‐adapted version, the Coma Recovery Scale for Pediatrics (CRS‐P), and showed that it provides reliable inter‐rater scores in typically developing (TD) infants and toddlers; however, normative and validation data in actual pediatric DoC populations remain scarce. A more recent study further suggested that, although the CRS‐R can detect longitudinal change in children aged 5–18 years after acquired brain injury, its sensitivity is reduced in the presence of severe motor impairment [2]. These diagnostic limitations are clinically significant because misclassification in pediatric DoC may distort prognosis, delay rehabilitation planning, and affect decisions about treatment intensity and family counseling. Recent reviews and guidelines underscore this gap: compared with adults, pediatric DoC lacks robust evidence‐based guidance for diagnosis, prognosis, and treatment, and no assessment modality has yet been shown to improve prognostic accuracy in children [3, 4]. On the therapeutic side, although pharmacologic interventions to promote arousal and consciousness recovery—most prominently amantadine—have gained empirical support in adults with DoC, the evidence in pediatric populations remains minimal [5]. A recent systematic update identified amantadine among the most promising agents for promoting recovery, yet nearly all available data derive from adult cohorts [6]. As a result, clinical care for children with DoC still relies largely on extrapolation from adult practice, small case series, and expert opinion. Given the high developmental stakes of early recovery, the limitations of existing behavioral tools, and the absence of pediatric‐specific therapeutic evidence, there is a compelling need to establish age‐appropriate assessment frameworks and rigorously evaluate interventions for pediatric DoC.

In the rehabilitation of DoC, several therapeutic strategies have been explored, including sensory stimulation, structured rehabilitation programs, noninvasive brain stimulation, and pharmacological neuromodulation. Among available pharmacologic options, amantadine is of particular interest because it is one of the few agents supported by controlled clinical evidence in DoC and remains the only pharmacologic treatment specifically recommended in adult practice guidelines for traumatic DoC [7, 8]. This makes it a particularly relevant candidate for pediatric investigation, where treatment decisions are still largely extrapolated from adult evidence. Amantadine acts both as a weak, uncompetitive antagonist of NMDA‐type glutamate receptors—thereby reducing excessive glutamatergic excitation—and as an indirect enhancer of dopaminergic neurotransmission, for example, by increasing dopamine release and reducing reuptake [9]. Preclinical data further suggest that amantadine may engage glial mechanisms, enhancing glutathione synthesis via astroglial cystine/glutamate antiporter (Sxc) activation while attenuating excitotoxic glutamate‐mediated damage [10]. From a neural‐circuit perspective, this pharmacology is especially relevant to DoC because dopaminergic and glutamatergic modulation may help restore function in cortico‐subcortical systems critical for wakefulness and awareness, particularly frontostriatal and thalamocortical loops [9]. Clinically, amantadine also stands out because, compared with many other candidate agents, it has the strongest empirical support in adult traumatic DoC, including evidence from a randomized, double‐blind, and placebo‐controlled trial demonstrating accelerated functional recovery during treatment [7, 8]. A more recent meta‐analysis similarly concluded that amantadine modestly improves short‐term consciousness and may hasten neurobehavioral recovery, although long‐term effects remain uncertain [11]. In addition, observational studies suggest potential benefit in nontraumatic acquired brain injury, including stroke and hemorrhage, with higher rates of early consciousness improvement than in untreated controls [12]. By contrast, although other therapeutic approaches for DoC are under active investigation, most have either weaker pharmacologic evidence, greater technical complexity, or even more limited pediatric data [13]. Thus, amantadine represents a rational and clinically relevant candidate for a pediatric study because it combines a plausible neurobiological mechanism, practical feasibility in routine care, and the most substantial evidence base currently available among pharmacologic interventions. However, despite growing evidence in adult DoC, its application in pediatric DoC remains largely unexplored, and recent reviews indicate that the pediatric literature is still dominated by observational studies and case reports, with no robust pediatric‐specific therapeutic framework currently available [4, 13]. This gap motivates the central question of the present study: whether amantadine is associated with measurable modulation of cortical oscillatory dynamics in pediatric DoC.

To investigate the neurophysiological effects of amantadine in pediatric DoC, a monitoring tool is needed that is objective, quantitative, repeatable, and feasible at the bedside. In this context, electroencephalography (EEG), particularly quantitative EEG (qEEG), is well suited for pediatric DoC because it is noninvasive, portable, relatively inexpensive, and can be repeatedly acquired in medically fragile patients without requiring transport or prolonged immobilization, unlike functional MRI or PET. These practical advantages make EEG especially valuable for the longitudinal monitoring of treatment‐related brain changes in children with DoC [14]. Among qEEG approaches, resting‐state spectral analysis provides a particularly relevant framework because it captures spontaneous cortical dynamics without requiring task comprehension, active cooperation, or preserved motor output—factors that are often compromised in DoC. Prior work has shown that resting EEG in DoC is typically characterized by a shift toward slower oscillatory activity, with increased delta/theta activity and reduced alpha‐band activity, and that these spectral features are associated with the level of consciousness and clinical outcome [15–17]. Accordingly, the present study adopted spectral indices derived from the approach of Lechinger et al. [15], who demonstrated that the CRS‐R is strongly related to resting‐state spectral EEG measures, particularly ratios reflecting the balance between faster and slower frequencies. In that study, higher alpha/theta and related fast‐to‐slow frequency ratios were associated with better behavioral responsiveness, supporting the view that frequency‐balance measures may provide clinically meaningful markers of residual brain function in DoC [15]. This framework is also consistent with broader qEEG literature, indicating that the relative preservation of faster rhythms and the reduction of pathological slow activity are among the most robust electrophysiological signatures of higher consciousness states in DoC [15, 16, 18]. Based on this rationale, we quantified resting EEG using relative EEG amplitude (RelAmp) in the delta, theta, alpha, beta, and gamma bands, together with two frequency‐balance indices: the alpha–theta ratio (ATR) and the slow–fast frequency ratio (SFR). These measures were selected because they provide a physiologically interpretable summary of cortical oscillatory organization, ranging from the relative predominance of slow‐wave activity to the re‐emergence of faster rhythms associated with arousal and behavioral responsiveness. In the context of pharmacologic neuromodulation, such indices are especially useful because they can detect whether treatment is associated with a shift from pathological EEG slowing toward a more activated oscillatory profile. Thus, EEG spectral analysis offers a practical translational framework for tracking treatment‐related cortical dynamics in the pediatric DoC.

This study aimed to address the limited understanding of pharmacological modulation in pediatric DoC by examining the neurophysiological effects of amantadine using resting‐state EEG in a prospective case–control framework. Thirty children were enrolled, including 15 with prolonged DoC and 15 age‐ and sex‐matched TD controls. The DoC group received a 1‐month course of amantadine, with EEG recorded before and after treatment. This design enabled assessment of within‐subject changes in the DoC group while providing a developmental reference from TD children. We hypothesized that children with DoC would show altered resting‐state EEG spectral profiles at baseline and that, following amantadine treatment, these spectral indices would shift toward a pattern characterized by reduced slow‐wave predominance and greater faster‐frequency activity. The study therefore aimed to determine whether amantadine is associated with measurable changes in RelAmp and frequency‐balance indices in pediatric DoC and whether these changes relate to behavioral responsiveness.

## 2. Method

### 2.1. Participants and Clinical Characteristics

A total of 30 children were enrolled in this study, including 15 children with DoC secondary to traumatic brain injury (TBI) and 15 age‐ and sex‐matched TD children. The DoC group comprised children aged 5–9 years (mean ± SD = 7.0 ± 1.8 years; 10 males and 5 females) who remained in a prolonged state of impaired consciousness 6–7 months after the injury, corresponding to the chronic stage of pediatric DoC. The TD group had no history of neurological, psychiatric, or developmental disorders and had achieved age‐appropriate developmental milestones. Children in the DoC group were recruited from Wuhan First Hospital and were diagnosed by experienced neurologists based on repeated clinical and behavioral assessments. Inclusion criteria for the DoC group were as follows: (1) a clinically confirmed diagnosis of prolonged DoC following TBI; (2) age between 5 and 9 years; (3) diagnosis established by experienced neurologists on the basis of repeated behavioral assessments using the CRS‐R at enrollment; (4) stable general medical condition permitting EEG assessment and pharmacological treatment; and (5) no severe visual or auditory deficits that would interfere with behavioral evaluation or EEG recording. Exclusion criteria included: (1) a history of epilepsy or other neurological disorders unrelated to the primary injury; (2) pre‐existing developmental, psychiatric, or neurological conditions unrelated to TBI; (3) unstable systemic illness; and (4) receipt of other investigational neurostimulatory interventions during the study period.

Diagnostic subgroup classification within the DoC cohort was determined according to standard CRS‐R diagnostic criteria based on item‐level evidence of conscious behavior rather than the total score alone. Among the 15 patients, 12 were classified as unresponsive wakefulness syndrome/vegetative state (UWS/VS) and three as minimally conscious state (MCS). Baseline CRS‐R total scores ranged from 6 to 17 and were used to characterize behavioral responsiveness rather than as a strict inclusion threshold.

Regarding the etiology, all patients in the DoC group had TBI. Routine clinical neuroimaging indicated extensive unilateral hemispheric injury in all patients, consistent with severe focal traumatic damage involving a large portion of one cerebral hemisphere. Importantly, the midline cortical regions selected for EEG analysis were not directly affected by focal traumatic lesions, which supported the use of midline electrodes for spectral analysis.

All children in the DoC group received oral amantadine for 1 month. The dosage was adjusted by body weight and administered as either 1.5–3 mg/kg every 8 h or 2.2–4.4 mg/kg every 12 h. Resting‐state EEG and behavioral assessment were performed at baseline (T0) and again immediately after the 1‐month treatment period (T1). Clinical follow‐up was further conducted at 3 months after baseline (T2), corresponding to a 2‐month post‐treatment follow‐up period. During both the treatment and follow‐up periods, all patients continued to receive standard supportive care and routine rehabilitation management according to their clinical need. Amantadine was administered only during the first month, and no additional experimental pharmacological or neuromodulatory interventions were introduced during the study period. All procedures were approved by the Ethics Committee of Wuhan First Hospital (Approval No. [2024]27), and written informed consent was obtained from the legal guardians of all participants in accordance with the Declaration of Helsinki.

### 2.2. EEG Data Acquisition

EEG recordings were performed using a Neurosoft EEG system (Neurosoft Ltd., Russia) with 24 Ag/AgCl electrodes positioned according to the international 10–20 system. The sampling rate was set to 1000 Hz, and signals were referenced to the linked earlobes during acquisition. The recordings took place in a quiet room with minimal electromagnetic interference. Each participant completed a 5 min resting‐state EEG session with eyes open, during which they were instructed to stay awake and minimize eye and body movements. Continuous EEG signals were band‐pass filtered between 1 and 40 Hz. Segments contaminated by gross artifacts (e.g., excessive muscle activity or movement) were visually inspected and manually removed.

### 2.3. Power Spectral Analysis of EEG

EEG spectral analysis was performed using MATLAB with the EEGLAB toolbox. Continuous EEG data were visually inspected and preprocessed prior to the spectral analysis. Bad channels were identified during preprocessing and were interpolated using EEGLAB’s spherical interpolation procedure when necessary. Importantly, none of the four electrodes included in the present region‐of‐interest analysis (Fz, Cz, Pz, and Oz) required interpolation. Nonstereotyped bad segments containing gross artifacts were removed following visual inspection. Independent component analysis (ICA) was then performed, and components were classified using EEGLAB’s built‐in component classification algorithm. Components showing at least 80% probability of being artifact‐related according to the EEGLAB built‐in classification algorithm were flagged for inspection, and clearly artifactual components were removed from the data.

The preprocessed continuous EEG data were then band‐pass filtered between 1 and 40 Hz using a zero‐phase finite impulse response filter to remove slow drifts and high‐frequency noise. The power spectral density (PSD) was estimated using Welch’s method implemented in the EEGLAB function spectopo, with 2‐s windows and 50% overlap. Following the spectral analysis procedure described by Lechinger et al. [15], EEG activity was quantified using RelAmp measures. For each electrode, the amplitude within each frequency band was calculated as the proportion of the spectral area within that band relative to the total spectral area between 2 and 40 Hz. This normalization approach reflects the contribution of each frequency band to the overall EEG spectrum and reduces variability related to recording conditions.

RelAmp was computed for five conventional frequency bands: delta (2–4 Hz), theta (4–8 Hz), alpha (8–12 Hz), beta (12–30 Hz), and gamma (30–40 Hz). In addition, two spectral ratios were derived to characterize the balance between slow and faster oscillatory activity: the ATR and the SFR, defined as the ratio between the combined alpha and beta activity and the combined delta and theta activity. All spectral metrics were calculated from four midline electrodes (Fz, Cz, Pz, and Oz), which are less susceptible to myogenic artifacts, and were averaged across these channels to obtain a single midline region‐of‐interest estimate for each participant. The following EEG spectral indices were included in the statistical analyses: relative delta, theta, alpha, beta, and gamma amplitude, as well as the ATR and SFR.

### 2.4. Data Statistics

All statistical analyses were performed using R version 4.3.1 [19]. Prior to inferential analyses, the distribution of all EEG spectral indices and behavioral measures was assessed for normality using the Shapiro–Wilk test, and the homogeneity of variance was examined using Levene’s test. All primary variables met the assumptions of normality (all *p* > 0.05) and variance homogeneity, allowing the use of parametric statistical methods. Baseline differences in neural activity between children with DoC and TD children were examined using a series of one‐way analyses of variance (ANOVAs) with group (DoC vs. TD) as the between‐subject factor. Dependent variables included RelAmp across five frequency bands (delta, theta, alpha, beta, and gamma), the ATR, and the SFR. Effect sizes were reported using partial eta‐squared (*η* ^2^). To evaluate the effects of the amantadine intervention, paired‐samples *t*‐tests were conducted within the DoC group to compare neural oscillatory metrics and clinical responsiveness measured by the CRS‐R between baseline (T0) and post‐intervention (T1, 1 month). Long‐term clinical recovery was further assessed by comparing CRS‐R scores at the 3‐month follow‐up (T2) with baseline values. Effect sizes for paired comparisons were calculated using Cohen’s *d*. To explore the association between neurophysiological measures and clinical outcomes, Pearson correlation analyses were performed between (1) baseline EEG indices and baseline CRS‐R scores and (2) early neurophysiological changes during treatment (ΔT1–T0) and long‐term behavioral recovery (ΔT2–T0). Given the relatively small sample size (*N* = 15) and the exploratory nature of these analyses, correlation results were interpreted cautiously in conjunction with overall group‐level trends. To control for multiple comparisons across frequency bands and regions of interest (ROI), the false discovery rate (FDR) was adjusted using the Benjamini–Hochberg procedure. Statistical significance was defined as FDR‐adjusted *p* < 0.05. All data are presented as the mean ± standard error of the mean (SEM).

## 3. Result

**Figure 1:**
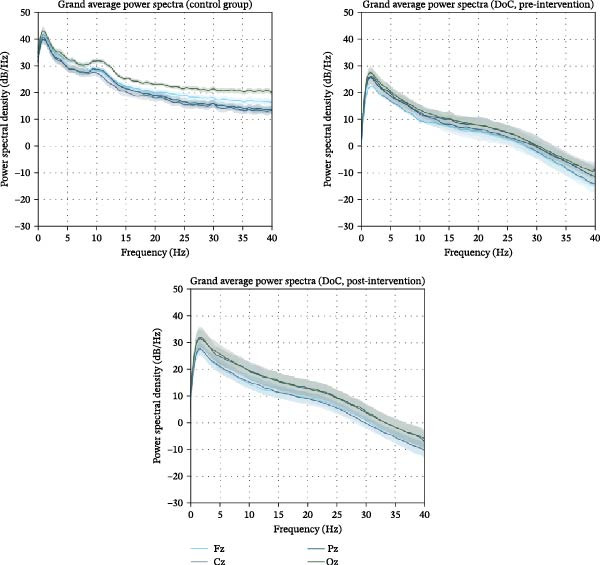
Grand‐average EEG power spectral density across groups.

Grand‐average PSD curves are presented in Figure 1, as a descriptive illustration of group‐level spectral characteristics. At baseline, children with DoC exhibited a spectrum with relatively greater low‐frequency activity and reduced alpha‐range activity compared with that of TD children. Following the amantadine intervention, the PSD curves suggest a modest shift in the spectral distribution. These visualizations are provided for descriptive purposes only, whereas all statistical analyses were based on predefined frequency‐band measures.

### 3.1. Baseline Group Differences in EEG Metrics

Baseline comparisons between children with DoC and TD revealed significant group differences in several EEG spectral indices (Table 1). Specifically, the ATR was significantly lower in the DoC group compared with TD children (*p* = 0.004, *η* ^2^ = 0.245). Similarly, the SFR was significantly reduced in the DoC group (*p* = 0.039, *η* ^2^ = 0.147). Regarding RelAmp, children with DoC showed significantly lower alpha amplitude (*p* = 0.003, *η* ^2^ = 0.240) and lower gamma amplitude (*p* < 0.001, *η* ^2^ = 0.314) compared with TD children. No significant group differences were observed for theta or beta amplitude (*p* > 0.05). A trend toward higher delta amplitude in the DoC group was observed, although this difference did not reach statistical significance (*p* = 0.054).

**Table 1:** Baseline EEG metrics in children with DoC and TD children.

Metric	DoC (T0) mean ± SE	TD children mean ± SE	*p* (FDR)	Partial *η* ^2^
ATR	0.337 ± 0.027	0.848 ± 0.137	0.004	0.245
SFR	0.284 ± 0.056	0.531 ± 0.086	0.039	0.147
Delta	0.576 ± 0.032	0.473 ± 0.035	0.054	0.126
Theta	0.290 ± 0.016	0.275 ± 0.010	0.510	0.021
Alpha	0.098 ± 0.011	0.205 ± 0.028	0.003	0.240
Beta	0.112 ± 0.018	0.112 ± 0.010	0.982	0.000
Gamma	0.005 ± 0.002	0.025 ± 0.004	<0.001	0.314

*Note:* Values are presented as mean ± SEM. *p*‐Values are adjusted using the Benjamini–Hochberg false discovery rate (FDR) procedure.

### 3.2. Correlation Between EEG Indices and CRS‐R Scores

**Figure 2:**
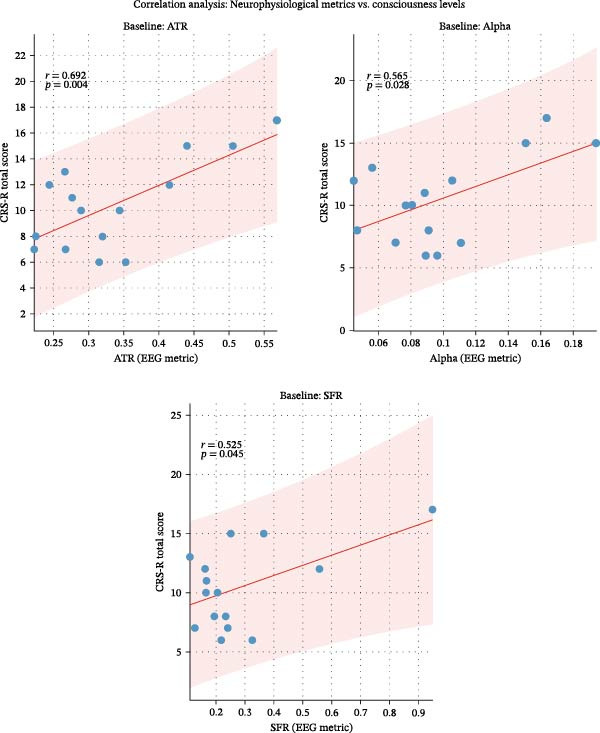
Correlations between EEG spectral indices and CRS‐R scores in children with DoC.

Pearson correlation analyses were conducted to examine the association between baseline EEG indices and behavioral responsiveness measured by the CRS‐R scores within the DoC group (Figure 2). Significant positive correlations were observed between ATR and CRS‐R scores (*r* = 0.692, *p* = 0.004) and between SFR and CRS‐R scores (*r* = 0.525, *p* = 0.045), indicating that higher ratios reflecting greater fast‐frequency activity were associated with better behavioral responsiveness. Among the spectral power measures, relative alpha amplitudes showed a significant positive correlation with CRS‐R scores (*r* = 0.565, *p* = 0.028). In contrast, delta, theta, beta, and gamma amplitude were not significantly correlated with CRS‐R scores (*p* > 0.05).

### 3.3. Effects of Amantadine on EEG Metrics

Following the amantadine intervention, significant changes were observed in several EEG spectral indices within the DoC group (Table 2). Specifically, the ATR increased significantly after treatment (*p* = 0.034, *d* = 0.701). Similarly, alpha power showed a significant increase (*p* = 0.036, *d* = 0.728). The most pronounced effect was observed in the gamma band, where relative gamma power increased significantly following the intervention (*p* = 0.005, *d* = 1.029). No significant changes were detected in SFR or in the relative power of the delta, theta, or beta bands (*p* > 0.05).

**Table 2:** Effects of amantadine intervention on EEG metrics in the DoC group.

Metric	Pre (T0) mean ± SEM	Post (T1) mean ± SEM	*p* (FDR)	Cohen’s *d*
ATR	0.337 ± 0.027	0.662 ± 0.127	0.034	0.701
SFR	0.284 ± 0.056	0.408 ± 0.086	0.264	0.378
Delta	0.576 ± 0.032	0.529 ± 0.040	0.266	0.348
Theta	0.290 ± 0.016	0.276 ± 0.011	0.528	0.195
Alpha	0.098 ± 0.011	0.163 ± 0.026	0.036	0.728
Beta	0.112 ± 0.018	0.098 ± 0.013	0.550	0.158
Gamma	0.005 ± 0.002	0.020 ± 0.003	0.005	1.029

### 3.4. Behavioral Outcome (CRS‐R)

Behavioral responsiveness assessed using the CRS‐R showed improvement following treatment. CRS‐R scores increased numerically from 10.5 ± 0.90 at baseline (T0) to 11.9 ± 1.20 after the intervention (T1), although this change did not reach statistical significance (*p* > 0.05). At the 2‐month follow‐up (T2), CRS‐R scores showed a significant improvement compared with baseline, increasing to 13.53 ± 0.80 (*p* < 0.001, *d* = 1.61), indicating substantial long‐term behavioral recovery.

### 3.5. Predictive Correlation Between Early EEG Changes and Long‐Term Recovery

To determine whether early neurophysiological changes predicted long‐term clinical recovery, Pearson correlation analyses were performed between EEG changes during the intervention period (ΔT1–T0) and CRS‐R improvement at the 2‐month follow‐up (ΔT2–T0). No significant correlations were observed between changes in ATR, SFR, or spectral power in any frequency band and long‐term CRS‐R improvement (*p* > 0.05).

## 4. Disscusion

The present study provides several key findings regarding resting‐state EEG spectral dynamics and treatment‐related changes in children with chronic DoC. First, compared with TD children, children with DoC secondary to TBI exhibited marked alterations in resting‐state EEG spectral profiles, characterized by reduced ATR, SFR, and relative alpha and gamma amplitude, together with a trend toward a higher delta amplitude. Second, at baseline, ATR, SFR, and relative alpha amplitude were positively associated with CRS‐R scores, supporting the relevance of these spectral indices to behavioral responsiveness in the pediatric DoC. Third, following a 1‐month course of amantadine, significant increases were observed in ATR, relative alpha amplitude, and relative gamma amplitude, indicating a shift toward faster cortical oscillatory activity. Notably, these EEG changes were evident immediately after treatment, whereas behavioral improvement was not significant until the 3‐month follow‐up, suggesting a temporal dissociation between neurophysiological change and overt clinical recovery.

The baseline EEG pattern observed in the present pediatric DoC cohort was characterized by reduced ATR, SFR, and relative alpha amplitude, together with a trend toward a higher delta amplitude, indicating a shift toward slower cortical oscillatory activity. This pattern is consistent with the broader literature showing that resting‐state EEG in DoC is typically marked by increased slow‐frequency activity and attenuation of faster rhythms, especially in the alpha range, reflecting impaired large‐scale cortical activation and reduced thalamocortical drive. From a physiological perspective, alpha‐band activity is closely linked to the integrity of thalamocortical loops and the maintenance of organized cortical processing, whereas a relative predominance of delta activity is generally interpreted as a marker of deafferentation, cortical dysfunction, or reduced arousal. In this sense, the present findings suggest that severe traumatic injury in these children may have disrupted the subcortical–cortical systems required to sustain normal fast oscillatory activity, thereby favoring pathological slowing [14, 20–22]. Importantly, our results also extend prior adult findings into a pediatric sample. Lechinger et al. [15] reported that in adult DoC, alpha/theta and related fast‐to‐slow spectral ratios were strongly associated with CRS‐R scores, indicating that the balance between faster and slower oscillations is clinically meaningful in DoC. The present study reproduces this general pattern in children, suggesting that ATR and related spectral indices may be robust markers of consciousness level across age groups, despite substantial developmental differences in baseline EEG organization. This point is particularly relevant because alpha rhythm matures across childhood, with peak alpha frequency increasing with age; thus, the pediatric EEG is physiologically slower than adult EEG even under normal conditions. Our data suggest that traumatic DoC does not simply preserve this immature profile but rather exaggerates slow‐frequency predominance beyond the expected developmental range, making ATR especially sensitive to the distinction between normal developmental immaturity and pathological cortical slowing [15, 18, 21, 23, 24]. A further notable finding was the large between‐group effect for the relative gamma amplitude. Although resting gamma activity in the DoC has been less consistently studied than alpha and delta rhythms, gamma‐band synchronization is generally considered to reflect local circuit‐level coordination and the capacity of cortical ensembles to sustain temporally precise neuronal interactions. The markedly reduced gamma amplitude observed here therefore may indicate a substantial loss of local cortical integration in the pediatric DoC, complementing the broader picture of reduced fast‐frequency organization. This interpretation should be made cautiously, given the known technical challenges of gamma‐band EEG, but the large effect size in the present data suggests that gamma abnormalities may carry additional information beyond conventional slow‐versus‐fast frequency shifts [16, 18, 22, 25].

The EEG changes observed after amantadine treatment can be interpreted within the framework of the anterior forebrain mesocircuit hypothesis, which proposes that severe brain injury reduces excitatory drive within cortico‐striato‐pallido‐thalamo‐cortical loops, leading to excessive inhibition of the thalamus and, consequently, diminished cortical activation. In this model, dopaminergic modulation may partially restore mesocircuit function by enhancing striatal output and reducing abnormal pallidal inhibition, thereby facilitating thalamocortical excitation and re‐engagement of cortical networks important for arousal and conscious processing [26, 27]. In this context, the significant post‐treatment increases in ATR and relative alpha amplitude are notable because alpha‐range activity is widely linked to the integrity of thalamocortical organization and the maintenance of coordinated cortical processing. The present findings, therefore, suggest that amantadine was associated with a partial shift away from pathological slowing toward a more activated oscillatory profile, consistent with the restoration of thalamocortical and frontostriatal network function [5, 28]. A particularly important aspect of the present results is the temporal dissociation between neurophysiological and behavioral recovery. EEG spectral changes were evident immediately after the 1‐month treatment period, whereas CRS‐R scores did not show significant improvement until the 3‐month follow‐up. This pattern is compatible with growing evidence in DoC research that covert or latent recovery of brain function may precede overt motor responsiveness. Recent work on cognitive motor dissociation (CMD) has shown that some behaviorally unresponsive patients retain measurable evidence of command‐following or higher‐order processing detectable by EEG or fMRI and that such covert consciousness is associated with more favorable recovery trajectories [29–32]. Although the present study did not directly test for CMD, the dissociation between early EEG improvement and delayed behavioral change suggests that amantadine‐related neurophysiological recovery may emerge before sufficient reorganization of motor output systems to affect bedside behavioral scores. In other words, pharmacological modulation may first enhance the neural substrates of arousal and cognitive processing, while observable behavioral recovery requires additional time and plasticity within the distributed networks necessary for motor expression and consistent command‐following. This interpretation is also consistent with prior mesocircuit models emphasizing that recovery of cognition and recovery of motor output can be partially dissociable after severe brain injury [26]. The marked increase in relative gamma amplitude after treatment, which showed the largest effect size among all EEG measures, may provide additional insights into this early neurophysiological shift. Gamma‐band activity has often been linked to local neuronal synchronization, temporally precise interactions between excitatory and inhibitory cortical circuits, and transient functional integration within cortical assemblies [33–35]. From this perspective, the post‐treatment gamma increase may indicate improved local cortical coordination accompanying a broader shift toward faster oscillatory dynamics. At the same time, gamma‐band EEG should be interpreted cautiously because of its susceptibility to technical and physiological confounds, and because its relationship to consciousness is still debated [33, 36]. Nevertheless, in the context of the concurrent increases in ATR and alpha amplitude, the gamma finding supports the possibility that amantadine was associated not only with reduced pathological slowing but also with partial restoration of faster oscillatory processes relevant to cortical integration.

These findings have several potential clinical implications. First, the present data suggest that resting‐state EEG spectral indices, particularly ATR and relative gamma amplitude, may serve as practical adjunct biomarkers for monitoring neurophysiological status and treatment‐related change in pediatric DoC, a population in which evidence‐based diagnostic and therapeutic guidance remains limited [4, 14, 18, 37]. Second, the observation that EEG changes emerged before significant behavioral improvement raises the possibility that qEEG may help identify children with early but behaviorally covert recovery processes, thereby complementing bedside assessment when overt motor responsiveness remains minimal. This interpretation is consistent with current work on covert consciousness and CMD, which highlights the value of neurophysiological measures in revealing residual brain function not captured by behavior alone [37, 38]. At the same time, several limitations should be acknowledged. The sample size was modest, which likely reduced power for the exploratory correlation analyses linking early EEG change to later behavioral recovery and limits generalizability. In addition, the open‐label, single‐arm design without a placebo control prevents firm causal attribution of the delayed CRS‐R improvement to amantadine alone because spontaneous recovery and the cumulative effects of standard rehabilitation cannot be excluded; this is particularly relevant given that controlled adult studies have shown accelerated recovery during active treatment, whereas longer‐term effects remain less certain [8, 12]. Future studies should therefore test these findings in larger, multicenter pediatric cohorts with controlled designs and extended follow‐up and should integrate EEG with complementary modalities such as fNIRS, which is portable and well tolerated in children, and DTI, which can characterize evolving white matter injury and recovery after pediatric TBI [39, 40]. Overall, the present study supports resting‐state EEG spectral measures as promising tools for characterizing pediatric DoC and tracking pharmacologically associated brain changes over time, while underscoring the need for more rigorous longitudinal validation.

Taken together, the present findings suggest that resting‐state EEG spectral dynamics may provide a useful window into neurophysiological alterations and recovery processes in the pediatric DoC. The observed shift toward slow‐frequency dominance, its association with behavioral responsiveness, and its partial change following amantadine are broadly consistent with the idea that the balance between slow and fast oscillatory activity is related to the functional state of large‐scale brain networks. From a scientific perspective, these results extend prior observations in adult populations to children, while also highlighting the need to consider developmental factors when interpreting EEG spectral measures. From a clinical perspective, the finding that EEG changes were detectable prior to significant behavioral improvement suggests that EEG measures may have potential as complementary tools for monitoring neurophysiological change over time. However, these interpretations should be considered preliminary and require confirmation in larger, controlled studies.

## 5. Conclusion

In conclusion, this study suggests that resting‐state EEG spectral indices can capture both baseline abnormalities and treatment‐related neurophysiological changes in children with chronic DoC. Amantadine was associated with a shift toward faster cortical oscillatory activity, while behavioral improvement emerged later during follow‐up. These findings support the potential value of EEG as an objective tool for monitoring recovery processes in the pediatric DoC.

## Author Contributions

**Mengqing Luo, Rui Sun, and Xuanhui Ren:** methodology, formal analysis, data curation, writing – original draft. **Juan Cheng, Meng Zhang, Ruihua Xu, Jun Tan, Jialei Ge, and Xu Zhang:** data collection, investigation, resources. **Huaqian Wang:** software, technical support, data preprocessing. **Yan Ma:** conceptualization, supervision, project administration. **Chong Lu:** conceptualization, methodology, writing – review and editing, supervision.

## Funding

This research received no specific grant from any funding agency in the public, commercial, or not‐for‐profit sectors.

## Disclosure

All authors have reviewed and approved the final manuscript. All scientific content, data analysis, interpretation, and conclusions were independently reviewed and verified by the authors.

## Conflicts of Interest

The authors declare no conflicts of interest.

## Data Availability

The datasets generated and/or analyzed during the current study are not publicly available due to ethical and privacy restrictions involving vulnerable pediatric participants. The data are available from the corresponding authors upon reasonable request, subject to approval by the institutional ethics committee.
